# Trends in the association between body mass index and blood pressure among 19-year-old men in Korea from 2003 to 2017

**DOI:** 10.1038/s41598-022-10570-9

**Published:** 2022-04-26

**Authors:** Hee Byung Koh, Ga Young Heo, Kyung Won Kim, Joohyung Ha, Jung Tak Park, Seung Hyeok Han, Tae-Hyun Yoo, Shin-Wook Kang, Hyung Woo Kim

**Affiliations:** 1 Department of Internal Medicine, College of Medicine, Institute of Kidney Disease Research, Yonsei University, 50-1, Yonsei-ro, Seodaemun-gu, Seoul, 03722 South Korea; 2 Department of Draft Physical Examination, Gyeonggi-Incheon Regional Military Manpower Administration, Suwon, Korea

**Keywords:** Risk factors, Epidemiology, Cardiovascular diseases, Hypertension

## Abstract

The strength of association between the body mass index (BMI) and blood pressure (BP) varies with population and time. Therefore, identifying the trends in BMI-BP association in adolescents can help predict the upcoming metabolic and cardiovascular disease burden. For this reason, from physical examination data collected from 2003 to 2017, a total of 5,133,246 Korean men aged 19 years were assessed for the annual trends and changes in the BMI-BP association. During the 15-year period, the mean BMI increased from 22.5 to 23.5 kg/m^2^, and the prevalence of obesity increased from 16.7 to 21.4%. Meanwhile, the mean systolic BP (SBP) decreased from 122.8 to 122.3 mmHg in the first year and gradually increased to 125.9 mmHg afterward. The diastolic BP (DBP) decreased from 71.5 to 70.0 mmHg in the first 4 years and then rose to 74.8 mmHg in the following years. The association analysis between BMI and SBP resulted in an annual increase in the correlation coefficient (SBP: 0.257–0.495, DBP: 0.164–0.413). The regression coefficient similarly increased between 2003 and 2015 but slightly decreased between 2015 and 2017 (SBP: 0.896–1.569, DBP: 0.405–0.861). The BMI-BP association increased over time (coefficient of the interaction term > 0, *P* < 0.001). Moreover, as the BMI increased, the annual increase in BP and BP per unit BMI also increased. In conclusion, this study emphasized a continuous shift towards obesity in BMI distribution and intensifying BMI-BP association over time in young men. Further research on factors affecting this BMI-BP association is needed to fully validate the potential applications of this hypothesis.

## Introduction

Obesity is a major cause of hypertension (HTN), diabetes mellitus (DM), and dyslipidemia, which are risk factors for cardiovascular diseases^1^. The body mass index (BMI) is one of the indicators that can be used to easily assess the degree of obesity and is positively correlated with blood pressure (BP)^2–4^. High BMI values in adolescence are associated with coronary heart disease, stroke, and mortality in adulthood^5,6^. Moreover, elevated BP in children or adolescents is associated with high pulse wave velocity, high carotid intima-media thickness, left ventricular hypertrophy, cardiovascular disease, and mortality in adulthood^7–10^. Therefore, identifying the trends in BMI-BP association in adolescents is important in predicting the upcoming burden of cardiovascular diseases and establishing a national health policy.

Interestingly, the strength of the BMI-BP association decreased over time in some studies^11–13^, whereas it increased in other studies^14^. Its association was still under debatable among previous studies because heterogeneous factors such as age, sex, and race could contribute to conflicting results. Fortunately, achieving homogeneity in the study population is plausible in South Korea, where all men are obliged to undergo a physical examination at the age of 19 years under the conscription system. Therefore, this study aimed to investigate the annual trends in BMI and BP and the changes in the BMI-BP association in 19-year-old men who underwent conscription examination in Korea for 15 years.

## Results

### Baseline characteristics of study participants

Table 1 shows the anthropometric data in each year. During the 15-year period, the mean BMI increased from 22.5 to 23.5 kg/m^2^, with the proportions of men with obesity and those with severe obesity increasing from 16.7 to 21.4% and from 4.7 to 8.7%, respectively. The mean systolic BP (SBP) gradually increased from 122.3 to 125.9 mmHg from 2004 to 2017, whereas the mean diastolic BP (DBP) decreased from 71.5 to 70.0 mmHg during the first 5 years and increased to 74.8 mmHg afterward. Between 2005 and 2017, the percentage of examinees with SBP ≥ 140 mmHg increased from 2.5 to 9.3%, DBP ≥ 90 mmHg increased from 1.1 to 4.9%, and SBP/DBP ≥ 140/90 mmHg increased from 1.0 to 4.5%.

**Table 1:** Baseline characteristics according to the year.

Year	No. of examinees	Height, m	Weight, kg	BMI, kg/m^2^	Underweight	Normal weight	Overweight	Obese	Severe obese
2003	*n* = 325,284	173.3 (5.7)	67.9 (12.3)	22.5 (3.8)	33,223 (10.2)	168,959 (51.9)	53,419 (16.4)	54,328 (16.7)	15,355 (4.7)
2004	*n* = 322,533	173.4 (5.7)	68.4 (12.5)	22.7 (3.8)	30,127 (9.3)	164,404 (51.0)	55,090 (17.1)	56,544 (17.5)	16,368 (5.1)
2005	*n* = 313,139	173.5 (5.7)	68.7 (12.6)	22.7 (3.8)	29,128 (9.3)	157,631 (50.3)	54,230 (17.3)	55,635 (17.8)	16,515 (5.3)
2006	*n* = 305,611	173.6 (5.7)	68.8 (12.6)	22.7 (3.8)	27,991 (9.2)	154,172 (50.4)	53,203 (17.4)	54,302 (17.8)	15,943 (5.2)
2007	*n* = 314,979	173.6 (5.7)	68.7 (12.5)	22.7 (3.8)	29,734 (9.4)	160,554 (51.0)	54,241 (17.2)	54,434 (17.3)	16,016 (5.1)
2008	*n* = 322,381	173.7 (5.7)	68.8 (12.7)	22.7 (3.9)	29,709 (9.2)	164,531 (51.0)	55,304 (17.2)	55,489 (17.2)	17,348 (5.4)
2009	*n* = 329,860	173.8 (5.7)	68.5 (12.7)	22.6 (3.9)	33,507 (10.2)	171,210 (51.9)	54,143 (16.4)	53,938 (16.4)	17,062 (5.2)
2010	*n* = 356,329	173.8 (5.7)	68.4 (12.7)	22.6 (3.9)	35,841 (10.1)	185,978 (52.2)	58,323 (16.4)	57,799 (16.2)	18,388 (5.2)
2011	*n* = 371,052	173.9 (5.7)	68.4 (12.8)	22.5 (3.9)	37,984 (10.2)	194,350 (52.4)	59,829 (16.1)	60,047 (16.2)	18,842 (5.1)
2012	*n* = 372,412	173.7 (5.7)	68.5 (12.9)	22.6 (3.9)	38,504 (10.3)	190,367 (51.1)	61,450 (16.5)	62,421 (16.8)	19,670 (5.3)
2013	*n* = 374,664	173.7 (5.8)	68.8 (13.3)	22.7 (4.0)	39,555 (10.6)	186,719 (49.8)	61,381 (16.4)	65,214 (17.4)	21,795 (5.8)
2014	*n* = 373,839	173.6 (5.8)	69.0 (13.4)	22.8 (4.1)	38,983 (10.4)	182,601 (48.8)	62,610 (16.7)	66,813 (17.9)	22,832 (6.1)
2015	*n* = 362,846	173.7 (5.8)	69.7 (13.8)	23.0 (4.2)	36,334 (10.0)	172,062 (47.4)	61,404 (16.9)	68,352 (18.8)	24,694 (6.8)
2016	*n* = 352,261	173.4 (5.8)	70.4 (14.3)	23.3 (4.4)	33,533 (9.5)	156,082 (44.3)	61,994 (17.6)	72,501 (20.6)	28,151 (8.0)
2017	*n* = 336,056	173.5 (5.8)	70.9 (14.7)	23.5 (4.5)	31,898 (9.5)	144,664 (43.0)	58,593 (17.4)	71,767 (21.4)	29,134 (8.7)
Total	*n* = 5,133,246	173.6 (5.7)	68.9 (13.1)	22.8 (4.0)	506,051 (9.9)	2,554,284 (49.8)	865,214 (16.9)	909,584 (17.7)	298,113 (5.8)

Year	No. of examinees	SBP, mmHg	DBP, mmHg	SBP ≥ 140 mmHg	DBP ≥ 90 mmHg	SBP/DBP ≥ 140/90 mmHg			
2003	*n* = 325,284	122.8 (13.2)	71.5 (9.3)	12,618 (3.9)	5,049 (1.6)	4,827 (1.5)			
2004	*n* = 322,533	122.3 (13.0)	71.4 (9.2)	9,409 (2.9)	4,392 (1.4)	4,094 (1.3)			
2005	*n* = 313,139	122.6 (12.5)	71.2 (9.0)	7,827 (2.5)	3,305 (1.1)	3,100 (1.0)			
2006	*n* = 305,611	123.0 (13.0)	70.8 (9.2)	11,304 (3.7)	4,769 (1.6)	4,638 (1.5)			
2007	*n* = 314,979	122.8 (12.9)	70.0 (9.1)	12,840 (4.1)	5,122 (1.6)	4,926 (1.6)			
2008	*n* = 322,381	122.8 (13.3)	70.2 (9.3)	16,706 (5.2)	7,922 (2.5)	7,575 (2.3)			
2009	*n* = 329,860	123.3 (13.6)	70.4 (9.4)	22,888 (6.9)	9,452 (2.9)	8,836 (2.7)			
2010	*n* = 356,329	123.9 (13.6)	71.1 (9.3)	27,736 (7.8)	11,706 (3.3)	10,710 (3.0)			
2011	*n* = 371,052	124.1 (13.2)	71.7 (9.1)	26,807 (7.2)	12,716 (3.4)	11,756 (3.2)			
2012	*n* = 372,412	124.4 (13.1)	72.4 (9.1)	27,930 (7.5)	13,514 (3.6)	12,354 (3.3)			
2013	*n* = 374,664	124.1 (13.4)	72.8 (9.2)	28,734 (7.7)	14,565 (3.9)	13,665 (3.6)			
2014	*n* = 373,839	123.9 (13.4)	72.7 (9.3)	28,031 (7.5)	14,865 (4.0)	14,165 (3.8)			
2015	*n* = 362,846	124.6 (13.4)	73.3 (9.1)	29,420 (8.1)	14,614 (4.0)	14,014 (3.9)			
2016	*n* = 352,261	125.9 (13.1)	74.4 (9.1)	31,830 (9.0)	16,627 (4.7)	15,560 (4.4)			
2017	*n* = 336,056	125.9 (13.2)	74.8 (9.1)	31,263 (9.3)	16,527 (4.9)	15,001 (4.5)			
Total	*n* = 5,133,246	123.8 (13.3)	72.0 (9.3)	325,343 (6.3)	155,145 (3.0)	145,221 (2.8)			

Continuous variables are expressed as mean and standard deviation, whereas frequency variables are expressed as absolute numbers and percentages.

*BMI* body mass index; *SBP* systolic blood pressure; *DBP* diastolic blood pressure; DM, diabetes mellitus.

The sequential changes in the mean SBP, DBP, and BMI are presented using piecewise linear regression in Supplemental Fig. 1. SBP showed an annual increase rate of 0.230 mmHg without a specific breakpoint. Meanwhile, DBP with an annual decrease rate of 0.396 mmHg at the beginning eventually changed to an upward rate of 0.509 mmHg starting at the breakpoint near 2007. BMI initially at a decrease rate of 0.003 kg/m^2^ changed to an increase rate of 0.224 kg/m^2^ at breakpoint near 2013.

### Changes in BMI distribution

To delineate the changes in BMI distribution over time, additional linear regression was performed to analyze the mean, standard deviation (SD), 5th percentile, and 95th percentile of BMI in each year (Supplemental Table 1). From 2003 to 2017, the mean and SD of BMI increased over time (annual increase rate of mean, 0.041; 95% confidence interval [CI], 0.014–0.068; annual increase rate of SD, 0.042, 95% CI, 0.028–0.057). In addition, the lower tail decreased, and the upper tail increased over time (annual decrease rate of 5th percentile, 0.012; 95% CI, 0.005–0.019; annual increase rate of 95th percentile, 0.125; 95% CI, 0.071–0.180). Based on the results of piecewise linear regression, the changes in BMI distribution before and after 2013 were separately analyzed. From 2003 to 2013, the SD of BMI increased by 0.017 per year (95% CI, 0.010–0.025). From 2013 to 2017, the mean BMI increased by 0.197 per year (95% CI, 0.131–0.263), with SD shifting toward the upper tail (annual increase rate of SD, 0.121; 95% CI, 0.082–0.161; annual increase rate of 5th percentile, 0.020; 95% CI, −0.017 to 0.057; annual increase rate of 95th percentile, 0.450; 95% CI, 0.300–0.600).

### Association between BMI and BP

Table 2 shows the correlation coefficients between BMI and BP in each year. However, the strength of correlation between BMI and SBP was weak in 2003–2004 (correlation coefficient, 0.257–0.287; all *P* < 0.001), but it gradually increased and reached a moderate intensity in 2005–2017 (correlation coefficient, 0.314–0.495; all *P* < 0.001). Similarly, although the significant but weak intensity of BMI-DBP correlation was observed in 2003–2008 (correlation coefficient, 0.164–0.299; all *P* < 0.001), it also gradually increased in a later period. When the significance of the difference in the correlation coefficients for consecutive years was tested using Fisher’s z-transformation, except in 2010–2011 and 2015–2016, a significant increase in correlation coefficient was identified over 12 years over 14 years (one-tailed *P* < 0.05). The association between BMI and BP in each year was also identified using linear regression (Supplemental Table 2). The regression coefficient of SBP and DBP between 2003 and 2015 increased from 0.896 to 1.569 and 0.405 to 0.861, then slightly decreased to 1.452 and 0.839 over the following two years.

**Table 2:** Pearson’s correlation coefficients between body mass index and blood pressure in each year.

	Year	No of examinees	r	95% CI	*P*-value
SBP	2003	*n* = 325,284	0.257	(0.254–0.260)	< 0.001
	2004	*n* = 322,533	0.284	(0.281–0.287)	< 0.001
	2005	*n* = 313,139	0.314	(0.311–0.317)	< 0.001
	2006	*n* = 305,611	0.347	(0.344–0.350)	< 0.001
	2007	*n* = 314,979	0.379	(0.376–0.382)	< 0.001
	2008	*n* = 322,381	0.392	(0.389–0.395)	< 0.001
	2009	*n* = 329,860	0.411	(0.409–0.414)	< 0.001
	2010	*n* = 356,329	0.427	(0.424–0.429)	< 0.001
	2011	*n* = 371,052	0.416	(0.413–0.419)	< 0.001
	2012	*n* = 372,412	0.437	(0.434–0.440)	< 0.001
	2013	*n* = 374,664	0.461	(0.459–0.464)	< 0.001
	2014	*n* = 373,839	0.473	(0.471–0.476)	< 0.001
	2015	*n* = 362,846	0.493	(0.490–0.495)	< 0.001
	2016	*n* = 352,261	0.484	(0.481–0.486)	< 0.001
	2017	*n* = 336,056	0.495	(0.492–0.497)	< 0.001
DBP	2003	*n* = 325,284	0.164	(0.161–0.168)	< 0.001
	2004	*n* = 322,533	0.193	(0.190–0.196)	< 0.001
	2005	*n* = 313,139	0.205	(0.202–0.208)	< 0.001
	2006	*n* = 305,611	0.246	(0.243–0.249)	< 0.001
	2007	*n* = 314,979	0.277	(0.274–0.280)	< 0.001
	2008	*n* = 322,381	0.299	(0.295–0.302)	< 0.001
	2009	*n* = 329,860	0.311	(0.308–0.314)	< 0.001
	2010	*n* = 356,329	0.323	(0.320–0.326)	< 0.001
	2011	*n* = 371,052	0.309	(0.306–0.312)	< 0.001
	2012	*n* = 372,412	0.324	(0.321–0.327)	< 0.001
	2013	*n* = 374,664	0.362	(0.359–0.365)	< 0.001
	2014	*n* = 373,839	0.374	(0.371–0.377)	< 0.001
	2015	*n* = 362,846	0.396	(0.393–0.399)	< 0.001
	2016	*n* = 352,261	0.398	(0.395–0.401)	< 0.001
	2017	*n* = 336,056	0.413	(0.410–0.416)	< 0.001

*95% CI* 95% confidence interval; *DBP* diastolic blood pressure; *SBP* systolic blood pressure.

The annual trends in SBP and DBP were identified using linear regression (Table 3). Annual increases in SBP by 0.230 mmHg and DBP by 0.264 mmHg were indicated in Model 1. The adjustment of BMI to determine its impact on BP trends resulted in the annual increase in SBP reduced to 0.174 mmHg, and the annual increase in DBP reduced to 0.234 mmHg as shown in Model 2. This means that BMI accounts for 24.3% ([0.230–0.174]/0.230) and 11.4% ([0.264–0.234]/0.264) of changes in SBP and DBP, respectively. By applying the interaction model adjusted by the interaction term of year × BMI, we further investigated the association of change in BMI and BP over time as described in Model 3. As the coefficient of the interaction term was significant with a positive value (SBP, 0.040; 95% CI, 0.039–0.041; DBP, 0.032; 95% CI, 0.031–0.032), the strength of the BMI-BP association increased over time.

**Table 3:** Linear regression of blood pressure on year and body mass index.

	Model 1^a^	Model 2^b^	Model 3^c^
**SBP**			
Year	**0.230 (0.228–0.233)**	**0.174 (0.171–0.176)**	**−0.753 (−0.767 to −0.739)**
BMI		**1.360 (1.357–1.363)**	**−80.05 (−81.26 to −78.85)**
Year*BMI			** 0.040 (0.039–0.041)**
R^^2^	0.006	0.174	0.176
**DBP**			
Year	**0.264 (0.262–0.266)**	**0.234 (0.232–0.236)**	**−0.491 (−0.501 to −0.481)**
BMI		**0.723 (0.721–0.725)**	**−62.94 (−63.81 to −62.06)**
Year*BMI			**0.032 (0.031–0.032)**
R^^2^	0.015	0.111	0.115

Boldface indicates statistical significance (*P* < 0.001).

^a ^Linear regression coefficient (95% CI) of BP on year.

^b ^Linear regression coefficient (95% CI) of BP on year and BMI.

^c ^Linear regression coefficient (95% CI) of BP on year, BMI and year × BMI.

*95% CI* 95% confidence interval; *BMI* body mass index; *BP* blood pressure; *DBP* diastolic blood pressure; *SBP* systolic blood pressure.

Tables 4 and 5 show the mean SBP and DBP values in each year according to BMI categories. As BMI increased, SBP (coefficient of SBP on year: 0.080–0.644 in Model 1), SBP per unit BMI (coefficient of SBP on BMI: 0.659–1.538 in Model 2), and the strength of the BMI-SBP association (coefficient of SBP on year × BMI > 0 in Model 3) increased in all examinees except the underweight group. Similarly, as BMI increased, DBP (coefficient of DBP on year: 0.066–0.538 in Model 1), DBP per unit BMI (coefficient of DBP on BMI: 0.345–1.042 in Model 2), and the strength of the BMI-DBP association (coefficient of DBP on year × BMI > 0 in Model 3) increased in all the BMI groups. In addition, the amount of annual increase in SBP and DBP was significantly greater in examinees with higher BMI (coefficient of SBP or DBP on year x BMI category > 0; *P* < 0.001).

**Table 4:** Mean systolic blood pressure and annual trend of systolic blood pressure by the body mass index group.

Year	Underweight	Normal weight	Overweight	Obese	Severe obese
2003	118.7 (12.6)	121.2 (12.5)	124.0 (12.6)	126.6 (13.1)	132.6 (15.6)
2004	117.8 (12.4)	120.5 (12.3)	123.2 (12.3)	126.2 (12.7)	132.9 (15.1)
2005	117.8 (12.0)	120.6 (11.8)	123.5 (11.7)	126.5 (11.8)	133.9 (14.3)
2006	117.8 (12.5)	120.7 (12.2)	124.0 (12.0)	127.6 (12.1)	136.2 (14.2)
2007	117.2 (12.4)	120.4 (12.1)	123.7 (11.8)	128.0 (11.8)	137.4 (12.8)
2008	117.2 (12.5)	120.1 (12.3)	123.6 (12.0)	128.1 (12.2)	138.6 (13.9)
2009	117.3 (12.5)	120.6 (12.4)	124.5 (12.3)	129.3 (12.5)	140.4 (14.3)
2010	117.6 (12.4)	121.1 (12.3)	125.1 (12.1)	130.0 (12.5)	141.8 (14.5)
2011	118.3 (12.3)	121.5 (12.0)	125.4 (11.8)	130.1 (12.2)	141.0 (14.2)
2012	117.9 (12.1)	121.6 (11.8)	125.7 (11.6)	130.3 (11.9)	141.4 (14.3)
2013	117.2 (12.7)	121.0 (11.8)	125.4 (11.5)	130.3 (12.1)	141.6 (14.0)
2014	116.7 (11.8)	120.7 (11.8)	125.0 (11.7)	130.1 (12.1)	141.5 (14.3)
2015	117.1 (11.7)	121.1 (11.6)	125.5 (11.4)	130.4 (11.7)	142.2 (14.3)
2016	118.3 (11.7)	122.1 (11.6)	126.4 (11.2)	131.0 (11.4)	141.5 (13.0)
2017	118.1 (11.8)	122.0 (11.5)	126.2 (11.3)	130.9 (11.4)	141.4 (13.2)
Model 1^a^					
Year	**−0.029 (−0.036 to −0.021)**	**0.080 (0.076–0.083)**	**0.211 (0.205–0.217)**	**0.353 (0.347–0.359)**	**0.644 (0.632–0.655)**
Model 2^b^					
Year	**−0.019 (−0.027 to −0.011)**	**0.077 (0.073–0.080)**	**0.209 (0.204–0.215)**	**0.341 (0.335–0.346)**	**0.575 (0.564–0.586)**
BMI	**0.659 (0.619–0.699)**	**1.131 (1.119–1.142)**	**1.300 (1.256–1.343)**	**1.532 (1.514–1.550)**	**1.538 (1.523–1.554)**
Model 3^c^					
Year	0.073 (−0.093 to 0.239)	**−0.762 (−0.820 to −0.704)**	**−0.869 (−1.112 to −0.627)**	**−0.831 (−0.941 to −0.722)**	**0.349 (0.231 to 0.467)**
BMI	11.290 (−7.829 to 30.400)	**−79.914 (−85.512 to −74.310)**	**−89.513 (−109.9 to −69.09)**	**−86.065 (−94.25 to −77.88)**	**−12.155 (−19.30 to −5.009)**
Year × BMI	−0.005 (−0.015 to 0.004)	**0.040 (0.038–0.043)**	**0.045 (0.035–0.055)**	**0.044 (0.040–0.048)**	**0.007 (0.003–0.010)**

Boldface indicates statistical significance (*P* < 0.001).

^a ^Linear regression coefficient (95% CI) of SBP on year by BMI category.

^b ^Linear regression coefficient (95% CI) of SBP on year and BMI by BMI category.

^c ^Linear regression coefficient (95% CI) of SBP on year, BMI, and year × BMI by BMI category.

*95% CI* 95% confidence interval; *BMI* body mass index; *SBP* blood pressure.

**Table 5:** Mean diastolic blood pressure and annual trend of diastolic blood pressure the by body mass index group.

Year	Underweight	Normal weight	Overweight	Obese	Severe obese
2003	70.8 (9.0)	70.6 (9.0)	71.4 (9.1)	73.0 (9.5)	77.5 (10.5)
2004	70.6 (9.0)	70.4 (8.8)	71.4 (8.9)	73.1 (9.2)	78.2 (10.3)
2005	70.2 (8.8)	70.2 (8.6)	71.0 (8.6)	72.7 (8.9)	78.1 (10.0)
2006	69.4 (9.0)	69.5 (8.8)	70.7 (8.7)	72.8 (8.9)	79.0 (9.9)
2007	68.4 (8.8)	68.6 (8.6)	69.9 (8.6)	72.4 (8.8)	79.0 (9.5)
2008	68.6 (9.0)	68.6 (8.7)	69.9 (8.7)	72.7 (9.1)	80.3 (10.1)
2009	68.7 (8.9)	68.8 (8.7)	70.4 (8.8)	73.3 (9.1)	81.1 (10.4)
2010	69.2 (8.8)	69.5 (8.6)	71.1 (8.6)	74.0 (9.1)	81.9 (10.2)
2011	69.9 (8.7)	70.2 (8.5)	71.8 (8.5)	74.6 (9.0)	81.9 (10.0)
2012	70.2 (8.7)	70.8 (8.4)	72.6 (8.4)	75.2 (8.8)	82.5 (10.1)
2013	70.1 (8.6)	70.9 (8.4)	72.9 (8.4)	75.9 (8.9)	83.4 (10.1)
2014	69.8 (8.5)	70.8 (8.5)	72.9 (8.6)	75.9 (9.0)	83.6 (10.1)
2015	70.2 (8.4)	71.3 (8.2)	73.4 (8.3)	76.3 (8.6)	84.0 (9.8)
2016	71.2 (8.3)	72.2 (8.3)	74.2 (8.3)	77.1 (8.5)	84.3 (9.3)
2017	71.4 (8.3)	72.4 (8.2)	74.4 (8.2)	77.3 (8.5)	84.7 (9.2)
Model 1^a^					
Year	**0.066 (0.061–0.072)**	**0.161 (0.159–0.164)**	**0.270 (0.266–0.274)**	**0.368 (0.364–0.372)**	**0.538 (0.530–0.547)**
Model 2^b^					
Year	**0.061 (0.056–0.067)**	**0.160 (0.158–0.163)**	**0.269 (0.265–0.274)**	**0.360 (0.356–0.364)**	**0.492 (0.484–0.499)**
BMI	**−0.361 (−0.390 to −0.332)**	**0.345 (0.337–0.353)**	**0.688 (0.656–0.720)**	**1.017 (1.004–1.030)**	**1.042 (1.032–1.053)**
Model 3^c^					
Year	0.005 (−0.114 to 0.123)	**−0.590 (−0.632 to −0.549)**	**−0.422 (−0.599 to −0.245)**	**−0.401 (−0.482 to −0.320)**	**0.461 (0.378–0.545)**
BMI	−6.893 (−20.562 to 6.772)	**−72.169 (−76.193 to −68.151)**	**−57.542 (−72.442 to −42.641)**	**−55.859 (−61.914 to −49.816)**	**−0.794 (−5.829 to 4.241)**
Year × BMI	0.003 (−0.004 to 0.010)	**0.036 (0.034–0.038)**	**0.029 (0.022–0.036)**	**0.028 (0.025–0.031)**	**0.001 (−0.002 to 0.003)**

Boldface indicates statistical significance (*P* < 0.001).

^a ^Linear regression coefficient (95% CI) of DBP on year by BMI category.

^b^ Linear regression coefficient (95% CI) of DBP on year and BMI by BMI category.

^c ^Linear regression coefficient (95% CI) of DBP on year, BMI, and year × BMI by BMI category.

*95% CI* 95% confidence interval; *BMI* body mass index; *DBP* diastolic blood pressure.

### Association between BMI and HTN

To further support the trend of association between BMI and BP over time with other confounding factors, validation analysis was performed with another survey data of the Korean Community Health Survey. Baseline characteristics according to each study year are presented in Supplementary Table 3. In logistic regression analysis, the annual increase in the prevalence of HTN was identified (odds ratio [OR], 1.10; 95% CI, 1.04–1.15) (Model 1) (Supplementary Table 4). Furthermore, adjusting BMI with the interaction term (year × BMI) resulted in a significantly positive value of the coefficient of the interaction term, suggesting the intensified BMI-HTN association over time (OR, 1.17; 95% CI, 1.02–1.13) (Model 2). This association remained even after adjusting for confounding factors including sex, alcohol, smoking, low salt diet, moderate physical activity, and diabetes (Model 3).

## Discussion

The data analysis of 19-year-old Korean men over a 15-year period showed that SBP and DBP increased by 3.1 and 3.3 mmHg, respectively, and the proportion of men with SBP/DBP of > 140/90 mmHg increased fourfold. The BMI increased only by 1.0 kg/m^2^, but the proportion of men with obesity expanded approximately by 30%. Although BMI accounted for only 24.3% and 11.4% of the changes in SBP and DBP over time, respectively, the strength of the association between BMI and BP increased over time. As the degree of obesity intensified, the annual increase in BP and BP per BMI unit tended to rise over time.

Recently, several studies have reported the inverse trend in increasing BMI with decreasing BP. Incremental administration of antihypertensive medications may have partly contributed to the discrepancy in BMI and BP changes over time. The use of antihypertensive medication weakens the BMI-BP association^2^. However, several studies have reported that the strength of the BMI-BP association gradually weakens regardless of antihypertensive medication use. In the German adult population, the mean BMI remained unchanged (27.0 kg/m^2^), with a slight increase in obesity prevalence in 1998 and 2008–2011. However, the mean SBP decreased from 129.0 to 124.1 mmHg, and the strength of the BMI-SBP association also decreased over time. This weakening trend was consistent after adjusting for age, antihypertensive medication use, alcohol intake, sports activity, and socioeconomic status^12^. Similar results were observed in a study involving adults in Seychelles, from 1989 to 2004: the mean SBP and DBP slightly decreased from 133/87 to 131/86 mmHg in men and from 127/82 to 124/81 mmHg in women, whereas the prevalence of treated HTN markedly increased. The mean BMI showed a sharp increase; however, the BMI-BP association weakened, regardless of hypertensive treatment^11^. Even in children aged 9–11 years who were unlikely to be affected by these medications, weakening of the BMI-BP association was reported. Notably, the BMI and BP levels simultaneously increased, but their association decreased over time^13^. These studies commonly noted that data on BMI are insufficient to predict the changes in BP levels, and environmental and socioeconomic factors should be considered when studying the BMI-BP association.

By contrast, our study showed that the strength of the BMI-BP association increased over time in the young male population of South Korea. Such an association was identified in two aspects: correlation coefficient and linear regression coefficient. Although the strength of correlation was weak in the early period, it gradually increased and reached a moderate intensity in the later period. This increase in the consecutive correlation coefficient was confirmed using Fisher’s transformation. The increase in the coefficient of linear regression of BP on BMI was confirmed by the positive interaction term. These results are notable because the 19-year-old examinees were rarely affected by the use of BP medications and other comorbidities. Similarly, from 1996 to 2014, adolescents aged 9–18 years in Hong Kong showed an intensified BMI-SBP association over time, despite the discrepancy between BMI and SBP^14^.

To understand the underlying reasons for changes in the BMI-BP associations over time, considering the effects of changes in BMI distribution is necessary. Some studies have shown that the association between BMI and BP varies according to the BMI^4,15^. The increase in BP per BMI unit and the risk of HTN were higher in the high BMI group. In this study, SBP showed a positive association with BMI in all BMI categories, including the underweight group (regression coefficient of SBP on BMI: 0.659–1.538, Table 4). Otherwise, DBP showed a positive association with BMI in all BMI categories except the underweight group (regression coefficient of DBP on BMI: 0.345–1.042, Table 5). As the BMI increased, the regression coefficient of BP on BMI increased. The increase in mean BMI and change in BMI distribution shifting to the upper tail may have influenced the change in the BMI-BP association. However, this hypothesis does not provide a clear explanation of the increasing the strength of the BMI-BP association observed in 2003–2013, in which the mean BMI decreased without a significant change. Other factors such as dietary sodium^16^, alcohol consumption^17^, smoking^18^, BP medications^2^, and physical activity levels^19^ may affect the association between BMI and BP.

### Limitations

This study has some limitations. First, the single measurement of BP could not provide an accurate reflection of real BP. When the initial BP measured using an electronic sphygmomanometer exceeded 140/90 mmHg, a repetitive measurement was performed using a manual sphygmomanometer. This may have caused a measurement bias between examinees with BP level ≥ 140/90 mmHg and those whose initial BP level < 140/90 mmHg. Since the prevalence of high BP levels (≥ 140/90 mmHg) was higher in the later period, more examinees were likely affected by this measurement bias in the later period. Further studies on BP measurement under a standardized protocol are needed. Second, the afore-mentioned factors that may have affected the changes in BP were not considered in the main analysis. To overcome this limitation, a validation cohort was constructed with another representative population sample cohort. In validation analysis, the BMI-HTN association similarly intensified from 2009 to 2017 even after adjusting for confounding variables including sex, smoking, alcohol, physical activity, low salt diet, and diabetes. The independence of these well-known confounding factors from the relationship between BMI and BP is also supported by a report from a nationally representative survey of adolescents in Korea that the prevalence of smoking (from 27.0 to 17.7%), drinking (from 49.6% to 31.7%), and vigorous physical activity (30.4–41.7%) had been reduced from 2005 to 2017^20^. However, the prevalence of fast food and carbonated drinks consumption increased from 14.8 to 26.4% and from 30.3 to 43.0%, respectively, from 2009 to 2017. In this respect, the cohort or period effect from changes in the prevalence of confounders such as dietary habits could have influenced the BMI-BP association. Further studies are needed to determine whether these factors influence the BMI-BP association. Nevertheless, this study is considered significant because the BMI-BP association in 19-year-old Korean men was less affected by comorbidities and medication use. In addition, the same association tendency was confirmed in the sensitivity analysis of the examinees who were enlisted for active-duty service through physical examination (Supplemental Table 5). The active-duty service classification ensured the inclusion of a healthy population by excluding those with medical diseases such as resistant HTN, DM, chronic kidney disease, and cancer certified by the clinicians from 11 departments. Third, the association between BMI and BP may be nonlinear. We used simple linear regression for the intuitive interpretation of the coefficients, assuming linearity between BMI and BP. When additional polynomial regression was performed using the cubic term of BMI for non-linearity, we could identify an increasing association between BMI and BP (Supplemental Table 6). Fourth, in addition to the trend between BMI and BP, the paradoxical opposing trend in SBP and DBP was observed in 2003–2007, but we could not elucidate this phenomenon from this study. Finally, the causative interaction was difficult to assess using regression analysis from the pooled cross-sectional cohort.

In conclusion, with changes in the BMI distribution towards obesity, the strength of the association between BMI and BP increased in young men. With increasing evidence for the association between high BP in adolescents and subsequent cardiovascular disease in adulthood, early-life medical intervention to effectively reduce body weight and BP may help to ease the burden of cardiovascular disease in adulthood.

## Materials and methods

This study was conducted in accordance with the Declaration of Helsinki and approved by the Institutional Review Board of Yonsei University Health System (2021-2030-001). The requirement for informed consent was waived from the Institutional Review Board of the Yonsei University Health System due to the retrospective nature of the analysis. Data were obtained from an open government data portal in Korea (http://open.go.kr), and all personal information was anonymized (confirmation number for information disclosure: 5855556).

### Study population

In South Korea, the conscription system has been adopted since 1957, and all men are required to undergo a physical examination at age 19 to determine whether they are suitable for military service. Physical examination was conducted in 14 regional military manpower administrations under the annual plan for all 19-year-old men. Prior to the examination, the examinees were informed about the hospital records that they needed to submit. On the day of examination, a survey was conducted to determine their current medical conditions or any underlying disease. Clinicians reviewed the medical records and certified the physical grades according to the 411 classified diseases. Men with physical grades 1–3 are enlisted for active-duty service, those with grade 4 are enlisted for supplemental service, those with grade 5 are enlisted for the second citizen service, and those with grade 6 are exempted from military service. Initially 5,133,332 examinees classified with grades 1–6 from 2003 to 2017 were screened. Eighty-six examinees with missing data on BP, height, weight, and history of diabetes were excluded. In each year, fewer than 0.01% of examinees are excluded due to missing values. Finally, 5,133,246 examinees were analyzed (Supplemental Fig. 2).

### Anthropometric data measurement

Height, weight, BMI, and BP were assessed annually. BP was measured at the office by a trained nurse using a standardized protocol. After the examinees rested for 10 min in a sitting position, the SBP and DBP were measured. BP was measured using an electronic sphygmomanometer. If the BP level measured using an electronic sphygmomanometer exceeded 140/90 mmHg, a repeat measurement was performed manually. Height and weight were measured to the nearest 0.1 cm and 0.1 kg, respectively, using standardized methods with the examinees wearing the same light clothes. BMI was calculated by dividing body weight (kg) by height in meter squared (m^2^). BMI was categorized according to the Asia–Pacific region-specific BMI classification proposed by the International Obesity Task Force and World Health Organization Regional Office for the Western Pacific Region: underweight, < 18.5 kg/m^2^; normal, 18.5–22.9 kg/m^2^; overweight, 23–24.9 kg/m^2^; obese, 25–29.9 kg/m^2^; and severely obese, ≥ 30 kg/m^2^. All measurements were carried out in each regional military manpower administration in the same way by regulation and did not change during the study period.

### Statistical analyses

The significance of differences between the groups was compared using analysis of variance for continuous variables and Pearson’s chi-squared test for categorical variables. To visualize the secular trend in BMI and BP from 2003 to 2017, we plotted the mean BMI, SBP, and DBP against the measurement year. Piecewise linear regression was performed to identify the breakpoint at which the trend changes. The association between BMI and BP in each year was determined using Pearson’s correlation coefficient. The significance of the annual increase in the correlation coefficient was calculated using the following method. First, Fisher's transformation ($z{\text{r}}=\frac{1}{2}{\mathrm{log}}_{e}\left(\frac{1+r}{1-r}\right)$) was used to normalize the independent sampling distribution of coefficients, and the Z-score of the difference between the two correlation coefficients was calculated using the following formula ($z {\text{diff}}=\frac{{\text{z}}{\text{r1}}-z{\text{r2}}}{\sqrt{\frac{1}{N{1}-3}+\frac{1}{\sqrt{N{2}-3}}}}$), where N is the sample size for each year. Then statistical significance was determined as a *P* < 0.05, calculated from the Z-score by one-tailed. Simple linear regression was also used to fit the association between BMI and BP each year. To identify the trend of BP over time, all examinee data were included in the linear regression model with the year regarded as a predictor variable. The model was adjusted for BMI to identify the degree of explanation of BP trends by BMI. The degree of explanation of the BP trend by BMI was calculated as the change in the rate of annual increase of BP when BMI was added to the model including BP and year. We further evaluated whether the strength of the association between BMI and BP changed over time by adding the year × BMI interaction term to the model. A positive value in the interaction term indicates that the strength of the association between BMI and BP increases over time. To identify whether the BMI-BP association differed according to the BMI category, subgroup analysis was performed. In addition, to further identify whether the annual increase in BP differed according to the BMI category, an interaction analysis was performed with the linear regression model of BP by year. The significance of the difference in annual BP increase was determined by adding interaction term (year x BMI group categorical variable) to the model. We used simple linear regression for the intuitive interpretation of the coefficients, assuming linearity between BMI and BP. In addition, a polynomial regression model using the cubic term BMI was performed. A sensitivity analysis was also conducted in 4,438,485 examinees classified as grades 1–3 in active-duty service, after excluding 694,761 examinees classified as grades 4–6 who could be influenced by comorbidity and medication use. Finally, to further support the trend of the association between BMI and BP over time with other confounding factors, validation analysis was performed with another survey data of the Korean Community Health Survey. Detailed information about the Korean Community Health Survey and analytic method from this validation cohort were presented in the Supplemental Method. All statistical analyses were performed using Stata (Version 14.2; StataCorp LLC, Texas, USA) and R (version 3.6.1; www.r-project.org; R Foundation for Statistical Computing, Vienna, Austria).

## Supplementary Information

Supplementary Information.

## Data Availability

Physical examination data are available via Open Government Data portal in Korea. Researchers can request access through website application at (http://open.go.kr). Statistical codes used for analyses are available from Dr. Kim (e-mail, drhwint@yuhs.ac).
