# Supplementary material for: Trends in the association between body mass index and blood pressure among 19-year-old men in Korea from 2003 to 2017
Source: Sci Rep. 2022 Apr 26;12:6767. doi: 10.1038/s41598-022-10570-9 (PMC9043188; doi:10.1038/s41598-022-10570-9)

**SUPPLEMENTARY INFORMATION**

**Trend in the association between body mass index and blood pressure**

**among 19-year-old men in Korea from 2003 to 2017**

**Table of contents**

**Supplemental Method**

**Supplemental Reference**

**Supplemental Table**

**Supplemental Table 1** Linear regression of mean, standard deviation, 5th, and 95th percentile body mass index

**Supplemental Table 2** Linear regression of blood pressure on body mass index

**Supplemental Table 3.** Baseline characteristics according to year (Korean Community Health Survey)

**Supplementary Table 4**. Logistic regression of hypertension on body mass index (Korean Community Health Survey)

**Supplemental Table 5** Linear regression of blood pressure on year and body mass index in healthy population

**Supplemental Table 6** Polynomial regression of blood pressure on year and body mass index

**Supplemental Figure**

**Supplemental Figure 1.** Piecewise regression analysis of changes in mean systolic blood pressure, diastolic blood pressure, and body mass index

**Supplemental Figure 2.** Flow chart of the study population

**Supplemental Method**

*Validation cohort*

To further support the trend of the association between body mass index (BMI) and blood pressure (BP) over time with other confounding factors, validation analysis was performed with the Korean Community Health Survey. The Community Health Survey was conducted in 253 health centers across the country, targeting an average of 900 adults over the age of 19 in each city, county, through a two-step probability sampling. A detailed profile of the cohort was described previously.^1^ The purpose and method of the study were fully explained to the participants, and consent was obtained before proceeding with the study.

Initially, 22,777 participants aged 19 from 2009 to 2017 were screened. Three hundred thirty-two participants with missing data on height, weight, smoking, alcohol, physical activity, and dietary habit were excluded. Finally, 22,445 participants were analyzed. Hypertension (HTN) was defined as a questionnaire of “Have you ever been diagnosed with high BP by a doctor?”. Diabetes was defined as a questionnaire of “Have you ever been diagnosed with diabetes by a doctor?”. The moderate physical activity was defined as a vigorous physical activity for at least 20 minutes a day and three days a week for the past one week or moderate-intensity physical activity for at least 30 minutes a day and five days a week or more during the past one week. A low-salt diet was defined when one or more of the following questionnaire items were met: 1) When I eat food, "I eat a little bland." or "I eat very bland", 2) When I eat cooked food, "no added salt or soy sauce", 3) When I eat jeon, pancake, or tempura, "don't eat with soy sauce".

Logistic regression analysis was used to validate the trend association between BMI and HTN. A multistep logistic regression model was constructed for following purpose. In model 1, predictor variable was set as year to identify the trend of HTN over time. In model 2, BMI and other confounding factors including smoking, alcohol, physical activity, and low salt diet were included. In model 3, year × BMI interaction term was included to evaluate the change in strength of the association between BMI and HTN over time.

**Supplemental Reference**

1. Kang, Y. W. *et al.* Korea Community Health Survey Data Profiles. *Osong Public Health Res Perspect* **6**, 211-217. https://doi.org/10.1016/j.phrp.2015.05.003 (2015).

**Supplemental Table 1.** Linear regression of mean, standard deviation, 5th, and 95th percentile body mass index

| Year | Mean | SD | 5th percentile | 95th percentile |
| --- | --- | --- | --- | --- |
| 2003-2017 | **0.041 (0.014 to 0.068) ^**^** | **0.042 (0.028 to 0.057) ^**^** | **-0.012 (0.005 to 0.019) ^**^** | **0.125 (0.071 to 0.180) ^**^** |
| 2003-2013 | -0.003 (-0.022 to 0.015) | **0.017 (0.010 to 0.025) ^**^** | **-0.020 (-0.031 to -0.009) ^**^** | **0.033 (0.005 to 0.060) ^*^** |
| 2013-2017 | **0.197 (0.131 to 0.263) ^**^** | **0.121 (0.082 to 0.161) ^**^** | 0.020 (-0.017 to 0.057) | **0.450 (0.300 to 0.600) ^**^** |

***Note:*** Regression coefficient (95% CI) of representative values on year

Boldface indicates statistical significance (^*^*P*<0.05, ^**^*P*<0.001)

***Abbreviations:*** 95% CI, 95% confidence interval; SD, standard deviation

**Supplemental Table 2.** Linear regression of blood pressure on body mass index

|  | Year | Coefficient | S.E. | 95% CI | *P-*value |
| --- | --- | --- | --- | --- | --- |
| SBP | 2003 | 0.896 | 0.006 | (0.884 - 0.907) | **< 0.001** |
|  | 2004 | 0.966 | 0.006 | (0.955 - 0.977) | **< 0.001** |
|  | 2005 | 1.024 | 0.006 | (1.013 - 1.034) | **< 0.001** |
|  | 2006 | 1.175 | 0.006 | (1.164 - 1.187) | **< 0.001** |
|  | 2007 | 1.280 | 0.006 | (1.269 - 1.291) | **< 0.001** |
|  | 2008 | 1.348 | 0.006 | (1.338 - 1.359) | **< 0.001** |
|  | 2009 | 1.447 | 0.006 | (1.436 - 1.458) | **< 0.001** |
|  | 2010 | 1.496 | 0.005 | (1.486 - 1.507) | **< 0.001** |
|  | 2011 | 1.422 | 0.005 | (1.412 - 1.432) | **< 0.001** |
|  | 2012 | 1.463 | 0.005 | (1.453 - 1.472) | **< 0.001** |
|  | 2013 | 1.531 | 0.005 | (1.522 - 1.541) | **< 0.001** |
|  | 2014 | 1.557 | 0.005 | (1.548 - 1.566) | **< 0.001** |
|  | 2015 | 1.569 | 0.005 | (1.560 - 1.578) | **< 0.001** |
|  | 2016 | 1.445 | 0.004 | (1.437 - 1.454) | **< 0.001** |
|  | 2017 | 1.452 | 0.004 | (1.444 - 1.461) | **< 0.001** |
| DBP | 2003 | 0.405 | 0.004 | (0.396 - 0.413) | **< 0.001** |
|  | 2004 | 0.464 | 0.004 | (0.456 - 0.472) | **< 0.001** |
|  | 2005 | 0.479 | 0.004 | (0.471 - 0.487) | **< 0.001** |
|  | 2006 | 0.587 | 0.004 | (0.579 - 0.595) | **< 0.001** |
|  | 2007 | 0.657 | 0.004 | (0.649 - 0.664) | **< 0.001** |
|  | 2008 | 0.719 | 0.004 | (0.711 - 0.727) | **< 0.001** |
|  | 2009 | 0.752 | 0.004 | (0.744 - 0.760) | **< 0.001** |
|  | 2010 | 0.773 | 0.004 | (0.766 - 0.781) | **< 0.001** |
|  | 2011 | 0.729 | 0.004 | (0.722 - 0.736) | **< 0.001** |
|  | 2012 | 0.749 | 0.004 | (0.742 - 0.757) | **< 0.001** |
|  | 2013 | 0.824 | 0.003 | (0.818 - 0.831) | **< 0.001** |
|  | 2014 | 0.854 | 0.003 | (0.847 - 0.861) | **< 0.001** |
|  | 2015 | 0.861 | 0.003 | (0.854 - 0.867) | **< 0.001** |
|  | 2016 | 0.827 | 0.003 | (0.821 - 0.833) | **< 0.001** |
|  | 2017 | 0.839 | 0.003 | (0.833 - 0.845) | **< 0.001** |

***Abbreviations:*** 95% CI, 95% confidence interval; SBP, systolic blood pressure; DBP, diastolic blood pressure; SE, standard error

**Supplemental Table 3.** Baseline characteristics according to year (Korean Community Health Survey)

| Year | No. of  examinees | Height | Weight | BMI | HTN | DM | Current drinker | Current smoker | Low salt diet | Moderate physical activity |
| --- | --- | --- | --- | --- | --- | --- | --- | --- | --- | --- |
| 2009 | N=2,328 | 167.7 (8.4) | 60.2 (12.1) | 21.3 (3.3) | 15 (0.6%) | 4 (0.2%) | 1,310 (56.3%) | 262 (11.3%) | 1,527 (65.6%) | 462 (19.8%) |
| 2010 | N=2,322 | 167.7 (8.3) | 60.1 (12.0) | 21.3 (3.2) | 19 (0.8%) | 4 (0.2%) | 1,295 (55.8%) | 282 (12.1%) | 1,705 (73.4%) | 461 (19.9%) |
| 2011 | N=2,443 | 167.8 (8.5) | 60.2 (11.9) | 21.3 (3.2) | 26 (1.1%) | 2 (0.1%) | 1,502 (61.5%) | 320 (13.1%) | 1,855 (75.9%) | 470 (19.2%) |
| 2012 | N=2,500 | 167.4 (8.4) | 60.6 (12.2) | 21.5 (3.3) | 31 (1.2%) | 5 (0.2%) | 1,528 (61.1%) | 311 (12.4%) | 1,845 (73.8%) | 474 (19.0%) |
| 2013 | N=2,616 | 167.7 (8.5) | 61.0 (12.5) | 21.5 (3.3) | 35 (1.3%) | 11 (0.4%) | 1,679 (64.2%) | 336 (12.8%) | 1,952 (74.6%) | 556 (21.3%) |
| 2014 | N=2,701 | 167.9 (8.4) | 61.5 (12.3) | 21.7 (3.3) | 31 (1.1%) | 6 (0.2%) | 1,778 (65.8%) | 353 (13.1%) | 2,000 (74.0%) | 555 (20.5%) |
| 2015 | N=2,581 | 167.7 (8.4) | 62.0 (12.9) | 21.9 (3.5) | 36 (1.4%) | 9 (0.4%) | 1,714 (66.4%) | 355 (13.8%) | 1,892 (73.3%) | 529 (20.5%) |
| 2016 | N=2,539 | 167.3 (8.5) | 62.2 (13.3) | 22.1 (3.6) | 32 (1.3%) | 8 (0.3%) | 1,665 (65.6%) | 318 (12.5%) | 1,829 (72.0%) | 517 (20.4%) |
| 2017 | N=2,413 | 167.6 (8.6) | 62.8 (13.9) | 22.2 (3.8) | 43 (1.8%) | 9 (0.4%) | 1,533 (63.5%) | 290 (12.0%) | 1,790 (74.2%) | 531 (22.0%) |
| Total | N=22,443 | 167.6 (8.4) | 61.2 (12.6) | 21.7 (3.4) | 268 (1.2%) | 58 (0.3%) | 14,004 (62.4%) | 2,827 (12.6%) | 16,395 (73.1%) | 4,555 (20.3%) |

***Note:*** Continuous variables are expressed as mean and standard deviation, whereas frequency variables are expressed as absolute numbers and percentages.

***Abbreviations:*** BMI, body mass index; DM, diabetes mellitus; HTN, hypertension.

**Supplementary Table 4**. Logistic regression of Hypertension (Korean Community Health Survey)

|  | Model 1^a^ | Model 2^b^ | Model 3^c^ |
| --- | --- | --- | --- |
| Year | 1.10 (1.04-1.15) | 0.75 (0.60-0.95) | 0.72 (0.56-0.92) |
| BMI |  | 0.01 (0.01-0.01) | 0.01 (0.01-0.01) |
| Year × BMI |  | **1.01 (1.01-1.02)** | **1.02 (1.01-1.03)** |
| Sex |  |  | 0.30 (0.22-0.41) |
| Alcohol |  |  | 1.05 (0.80-1.38) |
| Smoking |  |  | 1.52 (1.14-2.04) |
| Low salt diet |  |  | 1.07 (0.81-1.41) |
| Moderate physical activity |  |  | 0.91 (0.68-1.21) |
| DM |  |  | 12.0 (5.77-26.7) |
| R^^2^ | 0.005 | 0.063 | 0.103 |

^a^ Logistic regression odds ratio (95% CI) of BP on year

^b^ Logistic regression odds ratio (95% CI) of BP on year, BMI, and year × BMI.

^c^ Logistic regression odds ratio (95% CI) of BP on year, BMI, year × BMI, alcohol, smoking, low salt diet, moderate physical activity, DM.

***Abbreviations:*** 95% CI, 95% confidence interval; BMI, body mass index; DM, diabetes mellitus.

**Supplemental Table 5.** Linear regression of blood pressure on year and body mass index in healthy population

|  | Model 1 ^a^ | Model 2 ^b^ | Model 3 ^c^ |
| --- | --- | --- | --- |
| SBP |  |  |  |
| Year | **0.334 (0.325 to 0.342)** | **0.214 (0.207 to 0.221)** | **-0.487 (-0.516 to -0.459)** |
| BMI |  | **1.415 (1.410 to 1.420)** | **-57.061 (-59.355 to -54.766)** |
| Year×BMI |  |  | **0.029 (0.028 to 0.030)** |
| R^^2^ | 0.015 | 0.121 | 0.123 |
| DBP |  |  |  |
| Year | **0.367 (0.362 to 0.373)** | **0.300 (0.295 to 0.305)** | **-0.165 (-0.185 to -0.145)** |
| BMI |  | **0.801 (0.797 to 0.804)** | **-37.942 (-39.590 to -36.293)** |
| Year×BMI |  |  | **0.019 (0.018 to 0.020)** |
| R^^2^ | 0.024 | 0.237 | 0.240 |

*Note:* Boldface indicates statistical significance (*P*<0.001)

^a^ Linear regression coefficient (95% CI) of BP on year

^b^ Linear regression coefficient (95% CI) of BP on year and BMI

^c^ Linear regression coefficient (95% CI) of BP on year, BMI and year × BMI

***Abbreviations:*** 95% CI, 95% confidence interval; BMI, body mass index; BP, blood pressure; DBP, diastolic blood pressure; SBP, systolic blood pressure

**Supplemental Table 6.** Polynomial regression of blood pressure on year and body mass index

|  | Model 1 ^a^ | Model 2 ^b^ | Model 3 ^c^ |
| --- | --- | --- | --- |
| SBP |  |  |  |
| Year | **0.230 (0.228 to 0.233)** | **0.171 (0.168 to 0.173)** | **-0.222 (-0.229 to -0.215)** |
| BMI |  | **0.381 (0.363 to 0.400)** | **0.489 (0.470 to 0.507)** |
| BMI^^2^ |  | **0.019 (0.019 to 0.020)** | **-1.445 (-1.469 to -1.421)** |
| Year×BMI^^2^ |  |  | **0.0007 (0.0007 to 0.0007)** |
| R^^2^ | 0.006 | 0.175 | 0.178 |
| DBP |  |  |  |
| Year | **0.264 (0.262 to 0.266)** | **0.229 (0.227 to 0.231)** | **-0.048 (-0.053 to -0.043)** |
| BMI |  | **-0.875 (-0.888 to -0.861)** | **-0.799 (-0.813 to -0.786)** |
| BMI^^2^ |  | **0.032 (0.031 to 0.032)** | **-1.001 (-1.018 to -0.984)** |
| Year×BMI^^2^ |  |  | **0.0005 (0.0005 to 0.0005)** |
| R^^2^ | 0.015 | 0.121 | 0.123 |

*Note:* Boldface indicates statistical significance (*P*<0.001)

^a^ Linear regression coefficient (95% CI) of BP on year

^b^ Linear regression coefficient (95% CI) of BP on year, BMI and BMI^^2^

^c^ Linear regression coefficient (95% CI) of BP on year, BMI, BMI^^2^ and year × BMI^^2^

***Abbreviations:*** 95% CI, 95% confidence interval; BMI, body mass index; BP, blood pressure; DBP, diastolic blood pressure; SBP, systolic blood pressure.

**Supplemental Figure Legends**

**Supplemental Figure 1.** Piecewise regression analysis of changes in mean systolic blood pressure, diastolic blood pressure, and body mass index


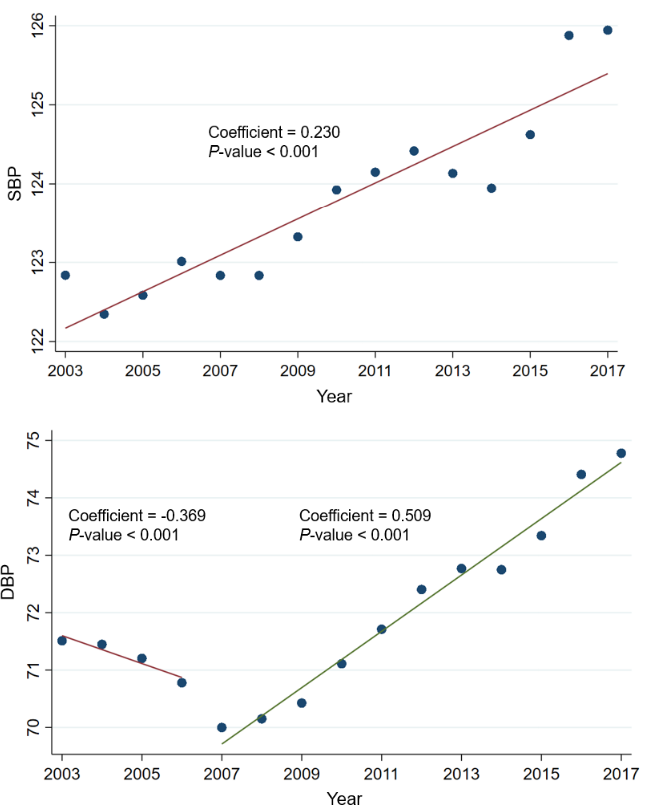


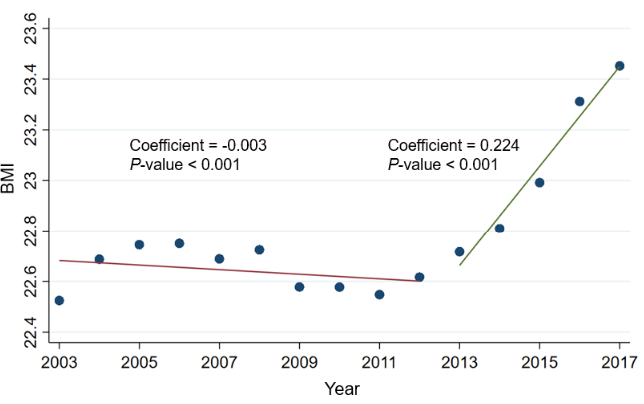


***Abbreviations:*** SBP, systolic blood pressure; DBP, diastolic blood pressure; BMI, body mass index.

**Supplemental Figure 2.** Flow chart of the study population


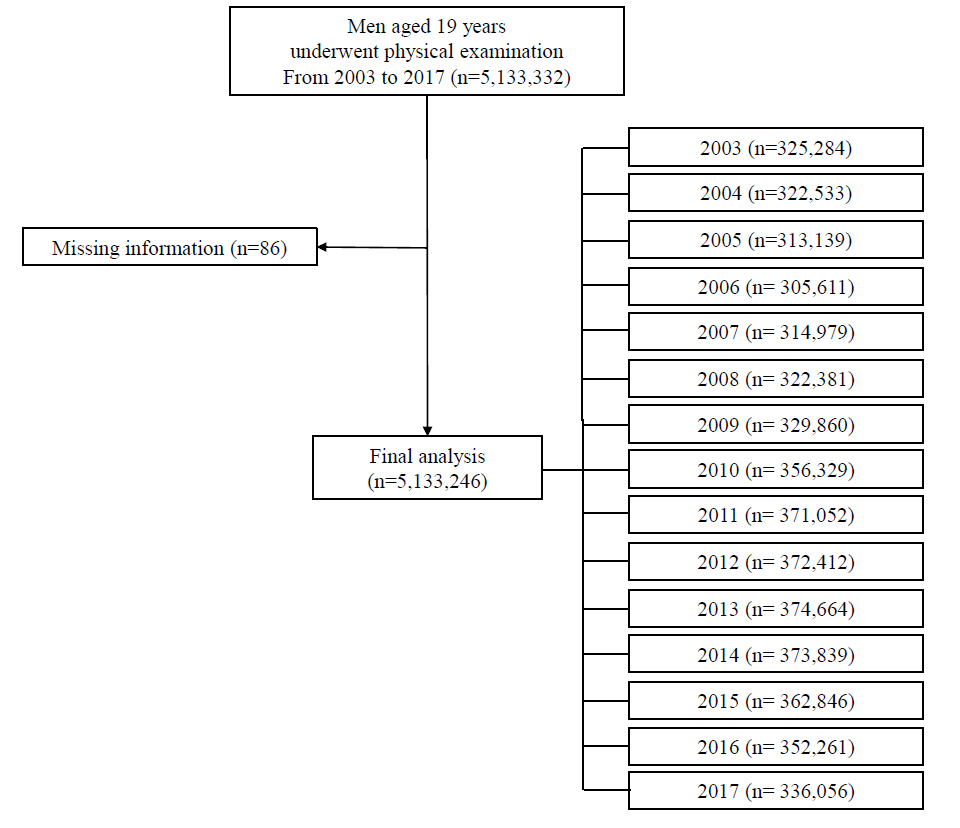

Supplement: Supplementary file 1 — Supplementary Information. [file 41598_2022_10570_MOESM1_ESM.docx]
